# Poggendorff rides again!

**DOI:** 10.1068/i0676sas

**Published:** 2015-01-06

**Authors:** Vebjørn Ekroll, Alan Gilchrist, Jan Koenderink, Andrea van Doorn, Johan Wagemans

**Affiliations:** Laboratory of Experimental Psychology, University of Leuven (KU Leuven), Leuven, Belgium, e-mail: vebjorn.ekroll@ppw.kuleuven.be; Psychology Department, Rutgers University, Newark, NJ, USA, e-mail: alan@psychology.rutgers.edu; Laboratory of Experimental Psychology, University of Leuven (KU Leuven), Leuven, Belgium; and Faculteit Sociale Wetenschappen, Psychologische Functieleer, Universiteit Utrecht, Heidelberglaan 2, 3584 CS Utrecht, The Netherlands, e-mail: Jan.Koenderink@ppw.kuleuven.be; Faculteit Sociale Wetenschappen, Psychologische Functieleer, Universiteit Utrecht, Utrecht, The Netherlands, e-mail: andrea.vandoorn@telfort.nl; Laboratory of Experimental Psychology, University of Leuven (KU Leuven), Leuven, Belgium, e-mail: Johan.Wagemans@psy.kuleuven.be

**Keywords:** adjustment, illusion, luminance gradient, occlusion

## Abstract

The Poggendorff illusion is one of the most exhaustively studied illusions. Can it be revived as an interesting problem? Perhaps by moving it to a slightly different domain. Here, we consider the occlusion of a subjectively linear ramp of tonal values. In a simple experiment, we find results closely resembling those of the geometrical Poggendorff. Yet, the “explanations” offered for the latter hardly apply to the former case. Depending upon one's perspective, this may be taken to “revive” the Poggendorff illusion.

Johann Christian Poggendorff (1796–1877) was not a vision scientist, but a German physicist mainly interested in electricity and magnetism. He pointed out the illusion in a drawing by the astronomer Zöllner (1860) in a letter he received as the editor of his(!) *Annalen der Physik und Chemie*. Thus, there is only a weak relation between the man and what we know as “The Poggendorff”.

The Poggendorff illusion has been researched in great detail (Burmester, 1896; Day & Dickenson, 1976; Fineman, 1996; Gillam, 1971, 1980; Greene 1988; Greene & Verloop, 1994; Greene & Fisher, 1993; Howe, Yang, & Purves, 2005; Lucas & Fisher, 1969; Masini, Sciaky, & Pascarella, 1992; Poulton, 1985; Spivey-Knowlton & Bridgeman, 1993). A review would be a voluminous, hardly “Short & Sweet”, but very instructive account of the scientific methods wielded in our field. One feels satisfied that the topic is closed.

As the authors happened to meet as lecturers at a summer course, sheer serendipity induced them to ask whether the Poggendorff might occur in different sections of the six-dimensional data volume (two spatial, one temporal, and three chromatic degrees of freedom). We decided on a position-luminance plane. Thus, we occluded a linear brightness ramp. Our question was whether the two visible legs of the ramp would reveal an illusory offset in brightness analogous to the offset in “height” characteristic of the traditional Poggendorff illusion.

There are some intricacies to deal with. For instance, the occluder and the background should not offer anchor points for the ramp. We decided to render the occluder in black–white texture, and the background deep blue (Figure 1) because neither can be comfortably matched to a uniform gray level. We also needed a subjectively linear (i.e., uniform) brightness scale, which we determined for each observer before the actual experiment. To provide ample freedom for brightness adjustments in any direction, we constrained the standard ramp to an intermediate range of gray values (64–191 from the device range 0–255). The task of the observers was to adjust the nominal brightness offset between the two half-ramps such that they appeared as a single linear brightness ramp partially hidden by the occluder. The offset was applied symmetrically around mid-gray; when the right ramp was brightened, the left ramp was darkened by the same amount (or vice versa). The perceptual offset experienced before manipulation of the physical offset is noticeable in Figure 1.

**Figure 1.**
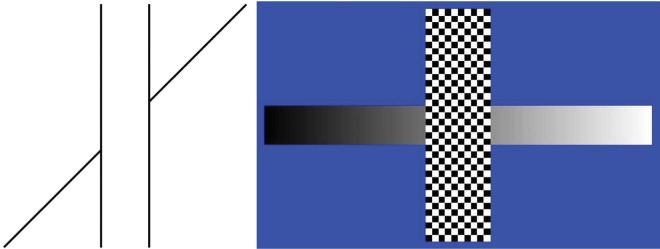
At left: The traditional Poggendorff illusion. A straight oblique line is occluded by a vertical outline “bar,” yet the right line segment appears “higher” than the straight extrapolation of the left line segment. At right: Demonstration of the “brightness Poggendorff.” Here, a linear brightness ramp is occluded by a vertical checkered bar. Most people experience the right side of the ramp as “brighter” than it should be if the part of the brightness ramp to the left of the occluder is linearly extrapolated. In order to avoid potentially confounding lightness anchoring or brightness contrast effects, we used a checkered “occluder” and a colored background with “indefinite brightness values.”

Each of the authors completed three cycles of brightness calibration and measurement of the “Poggendorff brightness offset.” In each of the calibration sessions, we estimated the mapping *f*(*x*) from device coordinates *x* to a linear (uniform) brightness scale by adjusting a linear arrangement of five achromatic square targets such that the brightness differences between any two neighboring squares were equal. The endpoints (“dark” and “bright”) of the scale were fixed. We fitted a third-order polynomial (*f*(*x*) = *a* + *bx* + *cx*^2^ + *dx*^3^) to the three sets of settings resulting from each calibration session and used this function to display linear brightness scales for the ramps displayed in the main experimental sessions. In the latter, clicking the “up” arrow raised the brightness (i.e., *f*(*x*)) of the entire ramp visible to the right of the occluder and simultaneously lowered the brightness of the entire ramp visible to the left of the occluder (and vice versa for the “down” button). This setting was done three times in each session.

The global average reveals a very significant “Poggendorff effect.” However, it is not quite clear-cut: Although four of the authors showed very similar results, one author had the opposite effect. This proved reproducible, thus we have to leave an open end here. In Figure 2, we show the result on omission of this participant, although there is hardly a visible difference with the full result. Anyway, the effect is huge (Michelson contrast 23 ± 13%), and a graph of the result shows great similarity to the original Poggendorff. Note that device intensity values (rather than “linear” brightness *f*(*x*)) are plotted on the *y*-axis of Figure 2 left. Plotted in terms of brightness, the curves would be straight rather than slightly curved.

**Figure 2.**
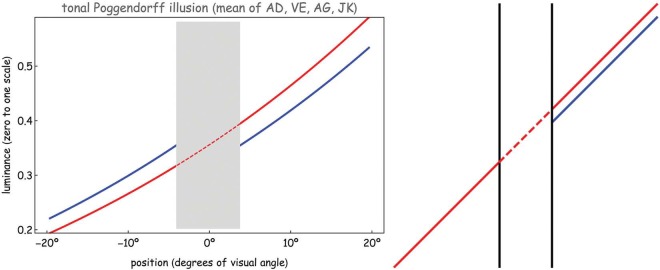
At left: The average of the three sessions by four of the authors (author JW had a qualitatively different effect, although this would hardly show up in the average). We used bilateral adjustment. The result looks much like a regular “Poggendorff illusion.” The *Y*-axis represents the 256 gray levels on the computer monitor rescaled to (0–1). The red curve (occluded part dashed) shows a grayscale ramp which appears as a linear brightness scale when it is not occluded. When occluded in the middle, however, the lower part appears too dark (or, equivalently, the upper part appears too light) for a subjectively linear brightness scale‥ The blue curves show the readjustment of the two visible parts of the brightness ramp necessary to correct for this illusion. At this setting, the two visible parts appear as a single linear brightness scale partially hidden behind the occluder. The curves shown here are the pointwise averages of the brightness ramps at the final settings chosen by the observers in terms of device gray values. At right: The geometrical Poggendorff in the same format, although with a (conventional) unilateral adjustment.

The explanations that have been offered for the Poggendorff illusion appeal to the intricacies of the perception of directions and angular relations. No such angular relations are present in our “tonal Poggendorff.” Nevertheless, the geometrical Poggendorff and our tonal Poggendorff are strikingly analogous, both in structure (an interrupted ramp) and in the percept. This strongly suggests a deeper explanation not specific to geometry or gray level. Our result certainly calls for further investigation: *Poggendorff rides again!*

## References

[R1] Burmester E. (1896). Beiträge zu experimentellen Bestimmung geometrisch-optischer Täuschungen. Zeitschrift für Psychologie.

[R2] Day R. H., Dickenson R. G. (1976). The components of the Poggendorff illusion. British Journal of Psychology.

[R3] Fineman M., Fineman M. (1996). Poggendorff's illusion.

[R4] Gillam B. (1971). A depth processing theory of the Poggendorff illusion. Perception & Psychophysics.

[R5] Gillam B. (1980). Geometrical illusions. Scientific American.

[R6] Greene E. (1988). The corner Poggendorff. Perception.

[R7] Greene E., Fisher J. (1993). Classical illusion effects with nonclassic stimuli: Angular induction from decomposing lines into point arrays. Perception & Psychophysics.

[R8] Greene E., Verloop M. (1994). Anomalous and luminance contours produce similar angular induction effects. Perception.

[R9] Howe C. Q., Yang Z., Purves D. (2005). The Poggendorff illusion explained by natural scene geometry. Proceedings of the National Academy of Sciences of the United States of America.

[R10] Lucas A., Fisher G. H. (1969). Illusions in concrete situations: II. Experimental studies of the Poggendorff illusion. Ergonomics.

[R11] Masini R., Sciaky R., Pascarella A. (1992). The orientation of a parallel-line texture between the verticals can modify the strength of the Poggendorff illusion. Perception & Psychophysics.

[R12] Poulton E. C. (1985). Geometric illusions in reading graphs. Perception & Psychophysics.

[R13] Spivey-Knowlton M. J., Bridgeman B. (1993). Spatial context affects the Poggendorff illusion. Perception & Psychophysics.

[R14] Zöllner, F. (1860). Ueber eine neue Art von Pseudoskopie und ihre Beziehungen zu den von Plateau und Oppel beschrieben Bewegungsphaenomenen. Annalen der Physik.

